# Supplementary figures and images for: The response of Dual-leucine zipper kinase (DLK) to nocodazole: Evidence for a homeostatic cytoskeletal repair mechanism
Source: PLoS One. 2024 Apr 4;19(4):e0300539. doi: 10.1371/journal.pone.0300539 (PMC10994325; doi:10.1371/journal.pone.0300539)

Fig 1 A

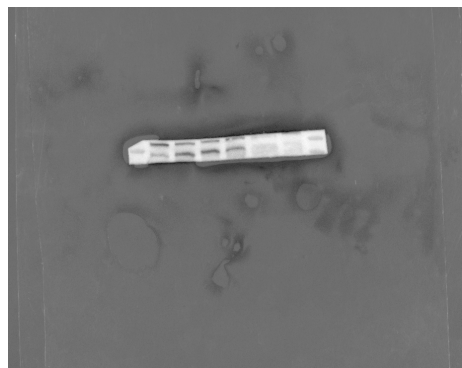

Phospho-MKK4

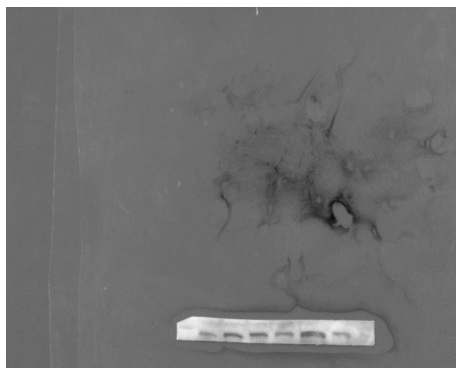

MKK4

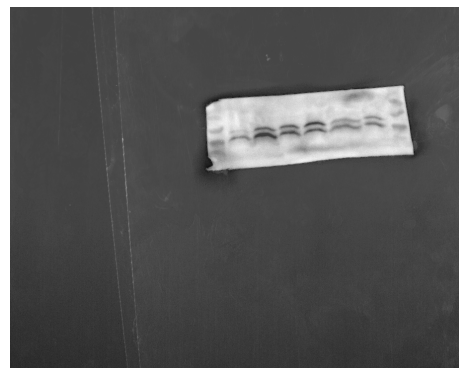

Histone H3

Fig 1 C

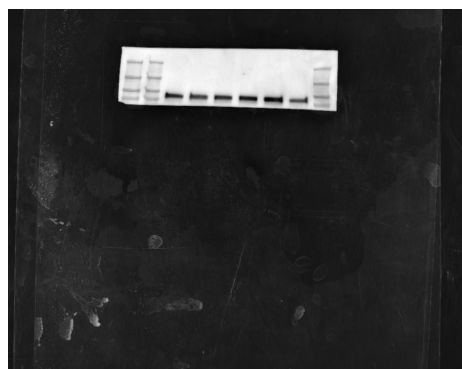

HSP90

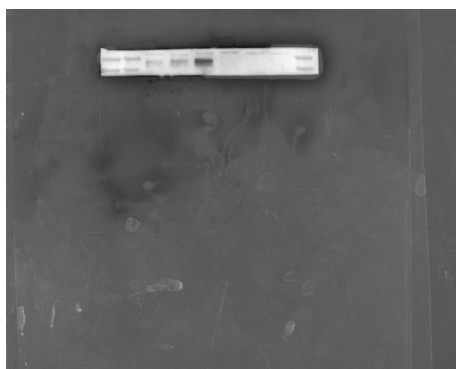

p-cjun

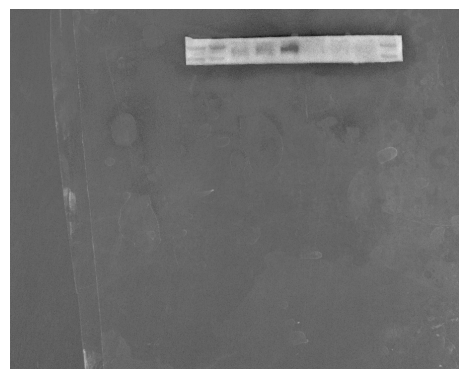

cJun

Supplement: S1 Raw images — (PDF) [file pone.0300539.s001.pdf]
