# Supplementary material for: The response of Dual-leucine zipper kinase (DLK) to nocodazole: Evidence for a homeostatic cytoskeletal repair mechanism
Source: PLoS One. 2024 Apr 4;19(4):e0300539. doi: 10.1371/journal.pone.0300539 (PMC10994325; doi:10.1371/journal.pone.0300539)

RSeQC: Infer Experiment

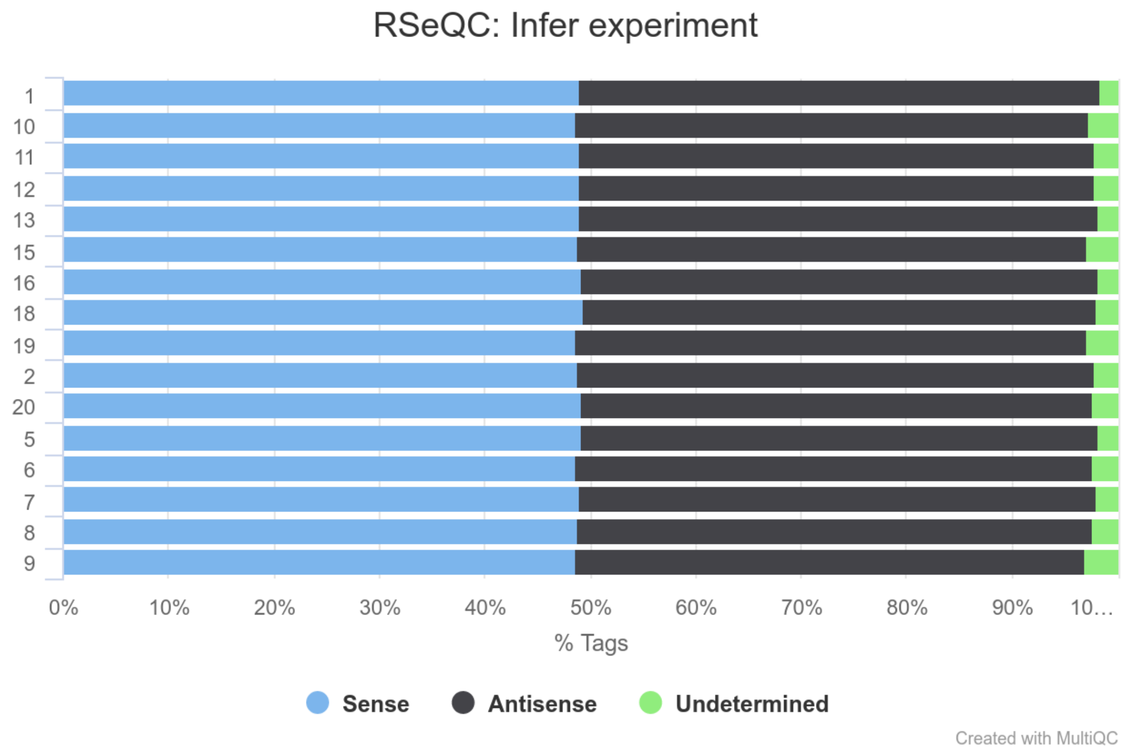

The libraries are unstranded

FastQC: GC Content (trimmed)

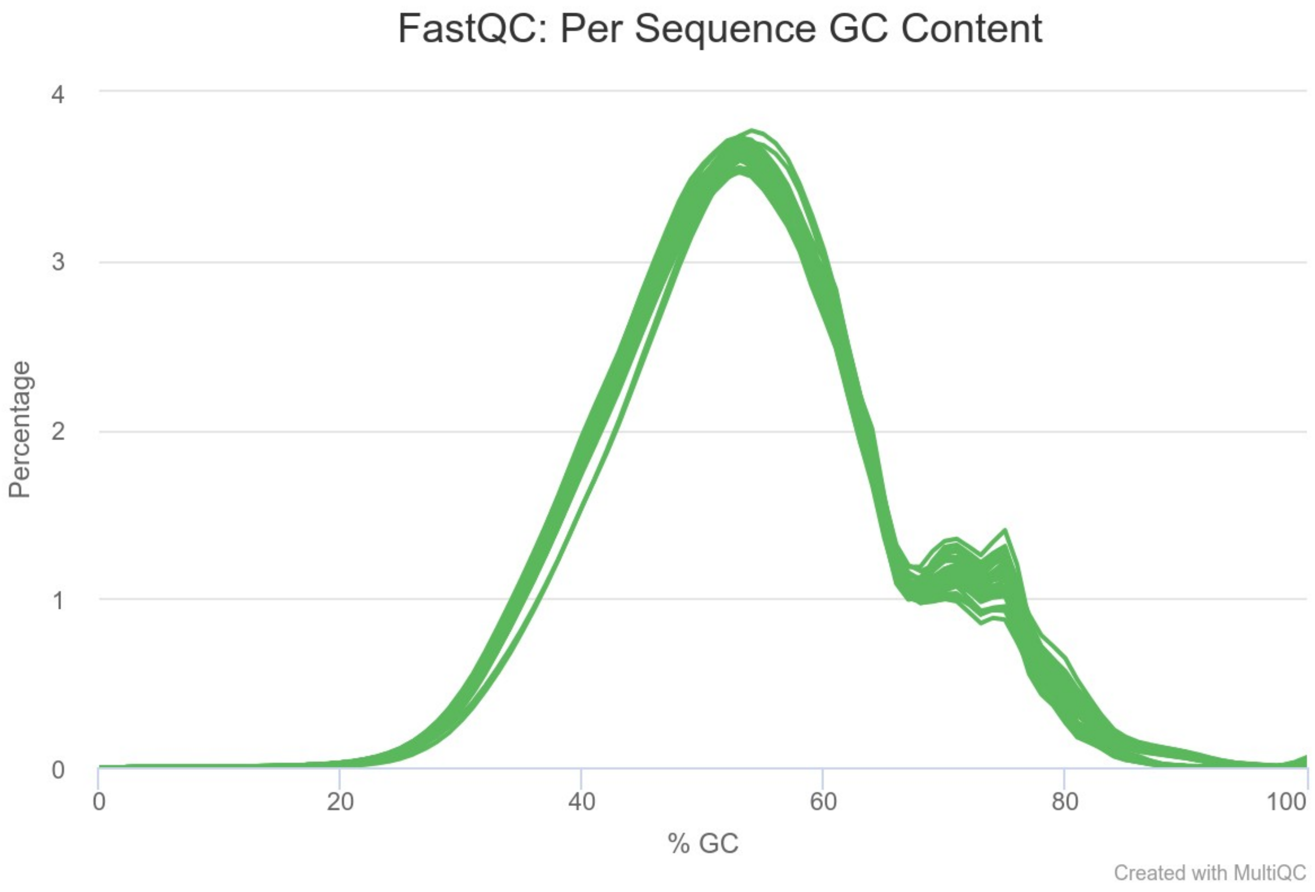

FastQC: Sequence Quality (trimmed)

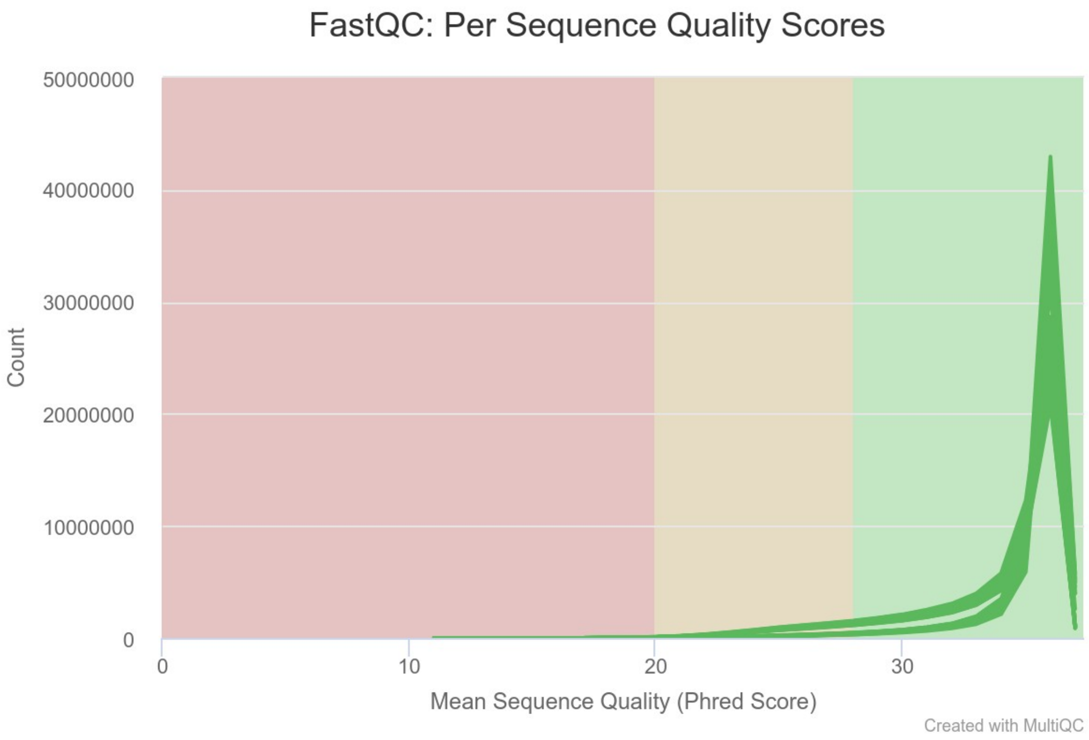

FastQC: Mean Base Score (trimmed)

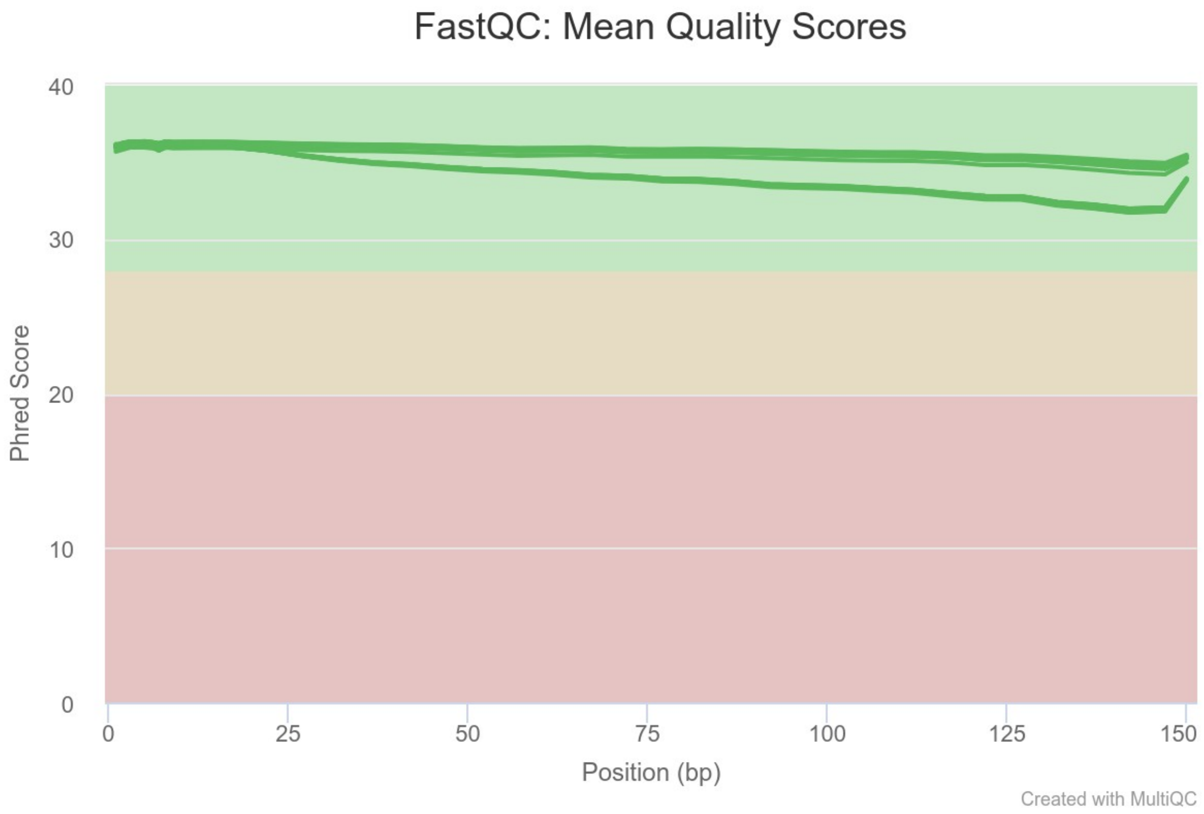

Picard: Duplication (percent of library)

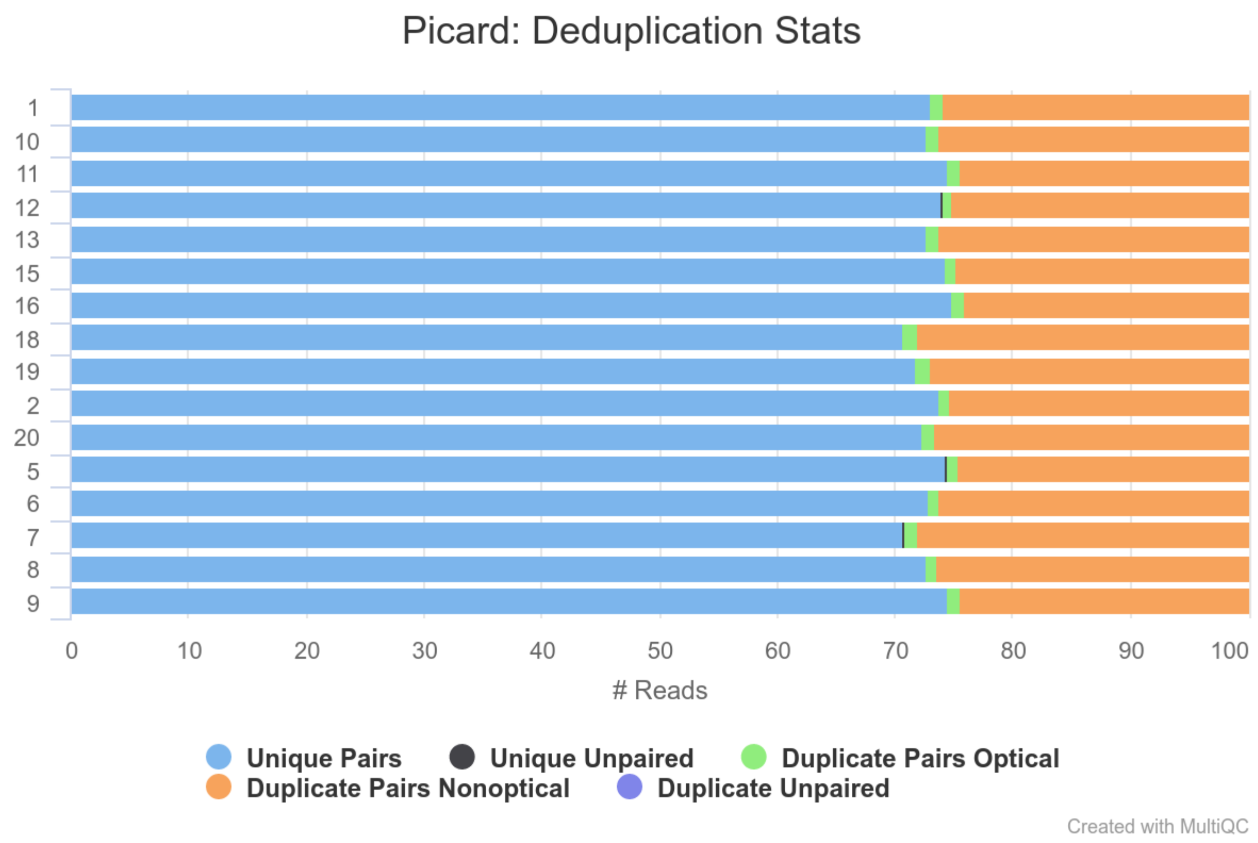

RseQC: Feature Distribution (percent of library)

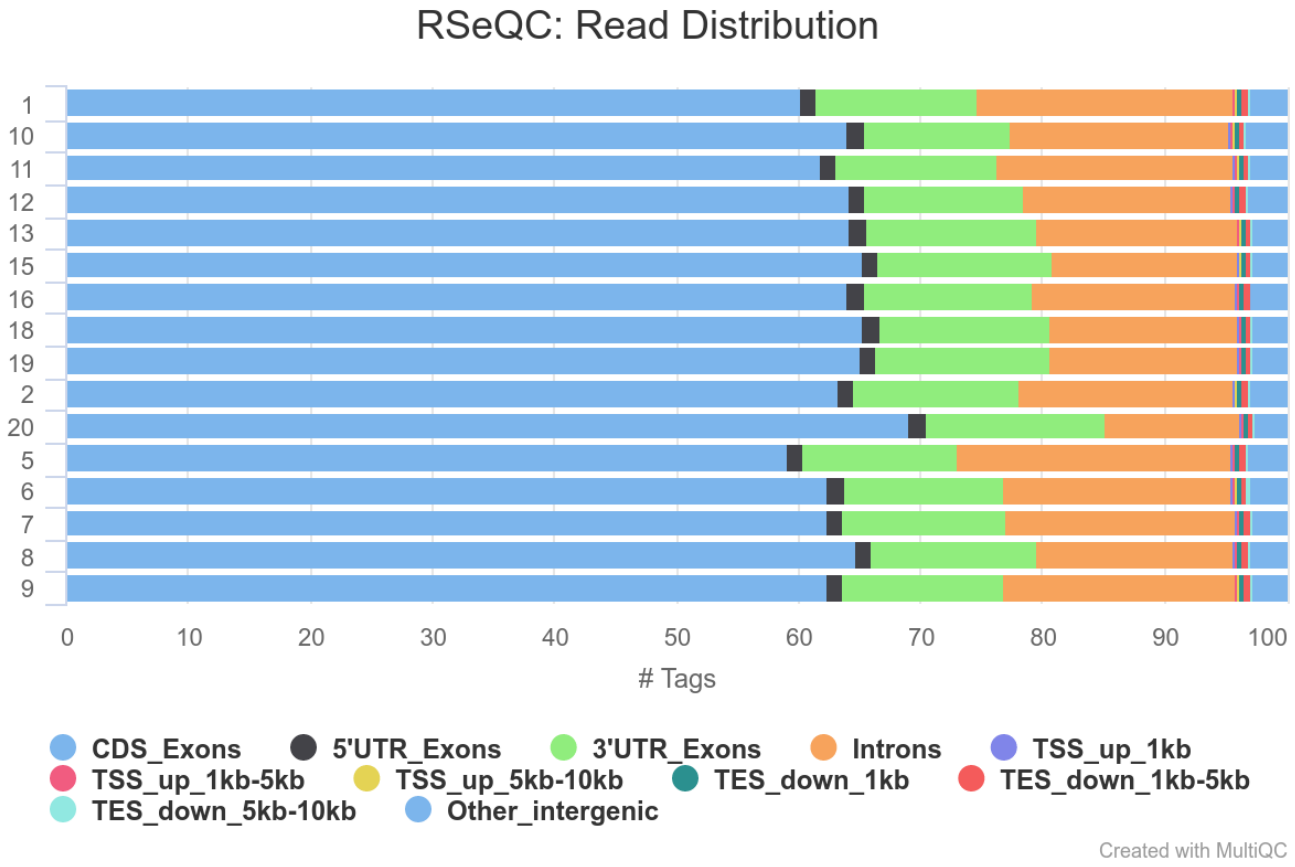

RseQC: Feature Distribution (raw counts)

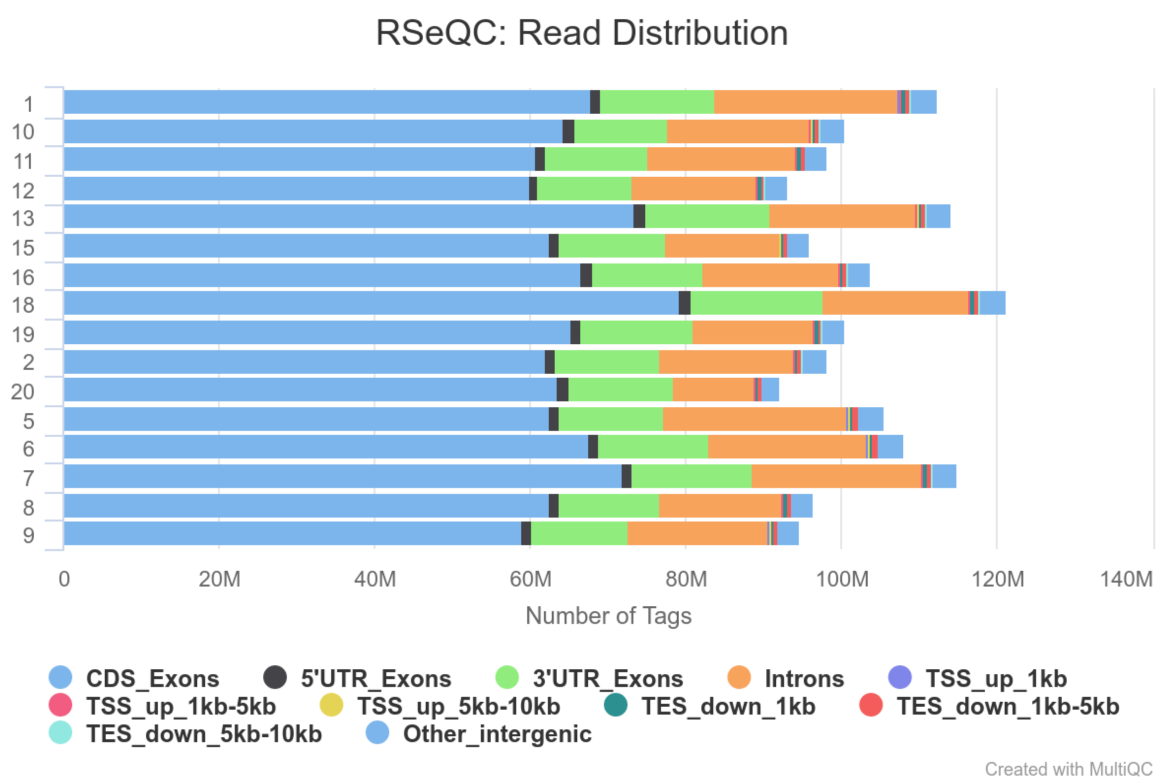

RseQC: Splice Junction Coverage (percent of library)

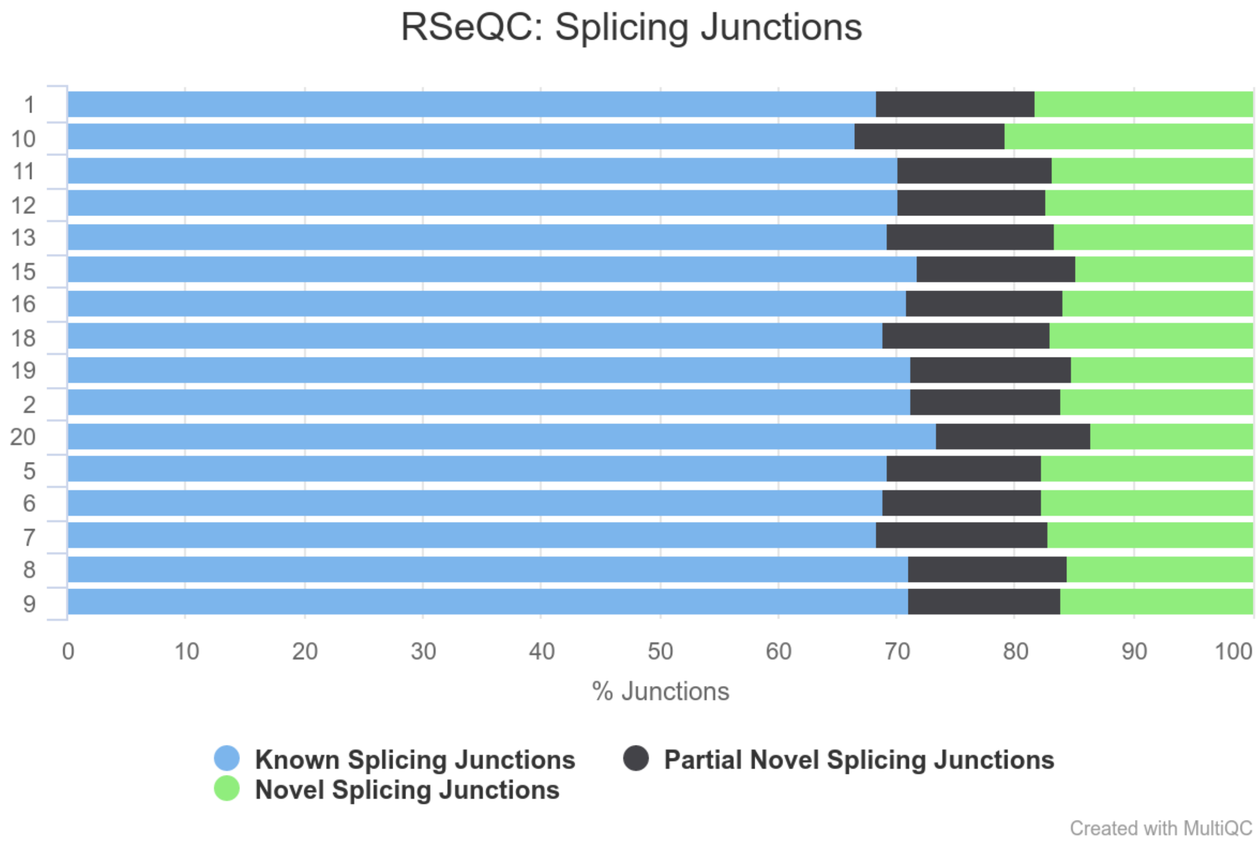

Qualimap: Gene Body Coverage

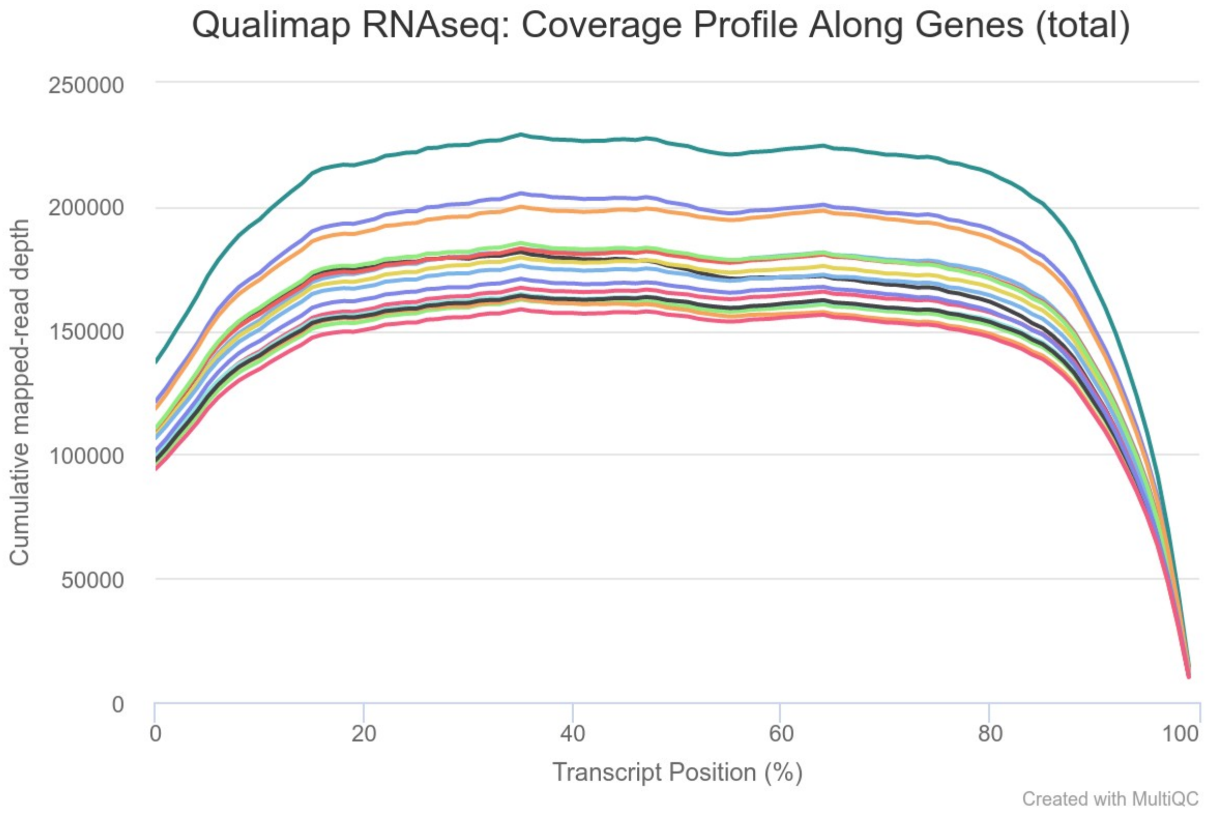

Supplement: S1 Fig — (PDF) [file pone.0300539.s004.pdf]
