# Supplementary material for: The response of Dual-leucine zipper kinase (DLK) to nocodazole: Evidence for a homeostatic cytoskeletal repair mechanism
Source: PLoS One. 2024 Apr 4;19(4):e0300539. doi: 10.1371/journal.pone.0300539 (PMC10994325; doi:10.1371/journal.pone.0300539)
Supplement: Attachment — Submitted filename: Response to reviewers.docx [file pone.0300539.s005.docx]

Response to reviewers:

We thank our reviewers for thoughtful consideration and commentary on our manuscript. We address all points raised by reviewers below.

1. Reviewer 1 (Comment 1) and Reviewer 2 (Comment 2) note that the blot in Fig 1 suggests that MKK4 levels are reduced upon DLK inhibitor treatment.

To assess the effect of DLK inhibitor treatment on MKK4 levels, we analyzed MKK4 across treatments, after normalization to a loading control (either HSP90 or Histone H3). We found that MKK4 levels are independent of DLK inhibitor treatment. Using a two-way ANOVA, we did not find a statistically significant difference between Noc (F=0.025, p = 0.875), DLKi (F=0.159, p=0.695) or the interaction of Noc and DLKi (F=0.029, p=0.867). Data are plotted below.

The apparent change in MKK4 levels demonstrated in the original Fig 1 were due to changes in the loading control. We have replaced the representative image with a more evenly loaded control (Histone H3). This Western better reflects MKK4 levels across treatments.

2. Reviewer 1 (Comment 2) notes that Jun is not detected in the DLK inhibition condition in either the untreated or 16H nocodazole treated neurons and thus differences in Jun cannot be differentiated.

DLK regulates the expression and phosphorylation of cJun. We quantify cJun protein levels (present at approximately 43 kDa, the molecular weight of cJun) and normalize this to the expression of HSP90, a loading control. After DLK inhibitor treatment, while the cJun band is rather faint at this exposure, cJun is measurable and quantifiable and so differences would be detectable.

While DLK inhibitor does lead to a large drop in cJun and pJun levels, this result is consistent with published results (Larhammar et al 2017A and B (PMID: 28993483; PMID: 28440222) that also studied the role of DLK in Jun regulation.

3. Reviewer 1 (Comment 2) notes that Fig 1B and Fig 1D should be switched.

Thank you for this suggestion. We switched Fig 1B and Fig 1D to match the quantification to the Western located on the left side of the quantification.

4. Reviewer 1 (Comment 3) notes that growth cone phenotypes are not quantified in Fig 1E.

We agree that this phenotype should be quantified and we did quantify this phenotype in Fig 4B, 4D. Figure 1 serves as an initial description of the phenotype.

5. Reviewer 1 (Comment 4) suggests that Fig 2B is a comparison of two samples and does not reflect the four groups used in the analysis.

The reviewer states that they did not find the text for the figure legend. The figure legend were previously provided on pg 11-13. In accordance with PLOS formatting guidelines, the Figure caption has been placed directed below the paragraph in which the figure is first mentioned. It is possible that if the review had found the textual figure legend, these concerns would have been addressed.

However, to further clarify the experiment design, we have added the following citation (<https://doi.org/10.1038/nmeth.3180>) and this text to the Results section:

“Using a two-factor completely crossed experimental design, we determined the transcriptional effects of nocodazole treatment, DLK inhibition and the interaction of nocodazole and DLK inhibitor.” Such an analysis does take into account all four conditions.

We have also added the following title to Figure 2B: “Principal Component Analysis” which may address the reviewer’s concern with this figure. Fig 2B is a PCA plot which demonstrates 4 treatment groups. These groups are identified by both color and shape in the legend in the figure itself. The textual figure legend additionally states this already.

The reviewer’s concern that there are only two groups in Fig 2B may be referring to Fig 2C. This figure is already titled “DLK-dependent effects of nocodazole”. The textual figure legend for Fig 2C already states the following “Differential gene expression in cultured DRGs of DLKi in low dose nocodazole. Log2FoldChange was determined in a two factor with interaction design in DESeq2”.

With the more explicit description of the experiment design in the Results section and a citation to a paper on two factor designs, the additional title on the PCA plot in Figure 2B, and the textual figure legend, we believe that Figure 2 is now clear.

We further now include a supplementary figure with the results of the DESeq2 analysis that derive from analysis of the other three groups (1) Effect of DLKi on Noc (2) Effect of DLKi and (3) Effect of Noc in S1_File.

6. Reviewer 1 (Comment 5) suggests that Fig 2D-F does not support a major claim and requests that the kinase-regulator activity in the Molecular Function term be redefined for MAPK activity.

To address this comment, we assessed the role of the MAPK cascade in our data set. We found that the GO term (GO:0000165 MAPK cascade) is significantly enriched in genes with a greater than one absolute value log2FoldChange. We have changed Figure 2C to highlight these genes. We have added the following to the figure caption:

Large changes in transcription factors (Egr1, Jun and Atf3), DUSP and MAPK cascade genes are noted in red, blue and yellow. MAPK cascade (GO:0000165) is significantly enriched in genes with a greater than 1 log2FoldChange (p = 1.6 x 10-9).

As the reviewer points out, GO enrichment is a standard analysis. We use it to broadly describe the effect of DLK-dependent cytoskeletal perturbation on the transcriptome. In particular, we use this analysis to suggest that in a DLK dependent manner in nocodazole treated neurons, DLK signaling promotes upregulation of transcription factors, signaling regulators and polarized growth programs and down regulation of mature neuron behaviors. These findings highlight statistically significant changes in neuronal growth state and maturity.

7. Reviewer 1 (Comment 6) suggests that Filopodial length, not number, is quantified in Fig 4.

In Fig 4C, Filopodia *per unit length* is quantified and compared. This measurement reflects the number of filopodia normalized to the axon length present in a given image and so is a measure of filopodia number as requested.

8. Reviewer 1 (Comment 7) notes that DLK inhibitor abolishes growth cones and suggests that a lower dose of DLK inhibitor might be informative. This concern is echoed in Comment 2.

Embryonic DRGs exhibit basal levels of DLK signaling (as evidenced by moderate levels of pMKK4/MKK4 and cJun in Fig 1). Previous work, including Oliva et al 2006 demonstrates that cJun and JNK signaling are required for axonal outgrowth. Through application of DLK inhibitor, we inhibit basal JNK and cJun signaling, thus impacting growth cones. To acknowledge this previous research, we’ve added the following text and citation to pg 7 line 201:

Within minutes of DLK inhibitor application, growth cones disappeared, consistent with the role of activated JNK signaling in axon outgrowth [(30)](https://www.zotero.org/google-docs/?8oCuNp).

[30.	Oliva AA, Atkins CM, Copenagle L, Banker GA. Activated c-Jun N-Terminal Kinase Is Required for Axon Formation. J Neurosci. 2006 Sep 13;26(37):9462–70.](https://www.zotero.org/google-docs/?EFTUZw)

Our observation that increased DLK signaling increases growth cones in a DLK-dependent manner is consistent with published literature demonstrating that conditioning lesions increase growth cones (Hammerlund et al 2009).

The dose of DLK inhibitor was selected based on previous literature (Larhammar et al 2017; Karney-Grobe et al 2018; Hayne et al 2018) as the minimal dose that robustly blocks DLK signaling.

9. Reviewer 1 (Comment 8) suggests that Figure Legends are absent from the manuscript.

Figure legends were previously provided on pg 11-13. In accordance with PLOS formatting guidelines, Figure captions are now placed below the paragraph in which the figure is first mentioned.

10. Reviewer 1 (Minor Comment 1) notes that MIG-2 is an important DLK activator and that Rac/Rho transcription may be changed after DLK signaling activation.

We do not observe dlk-dependent, nocodazole-dependent changes in MIG-2. We do observe minor noc-dependent, DLK-dependent increases in RhoC (log2fold 1.3), RhoV (1.2), RhoQ (1.2). While these changes are not individually noted, GO terms related to actin cytoskeletal organization are noted red in Fig 3B.

11. Reviewer 1 (Minor Comment 2) notes that single specificity serine/threonine phosphatases and tyrosine phosphatases could be important regulators of downstream signaling and suggests that it would be valuable to annotate in Fig 2B.

We note dlk-dependent, noc-dependent changes of ppp4r4 (l2foldchange 1.15), ppp1r21 (0.52), ppp2r3d (0.53), ppp1r15a (1.49), ppp2r5b (0.60), ptprn (0.6), ptprr (-1.3), ptpn23 (0.51), ptprt (0.93), ptpn4 (0.64), ptpn9 (0.52), ptpn5 (0.94), ptprk (-1.06), ptp4a3 (-0.89). These log2 fold changes are significant, but not among the greatest changes in our data set. These changes are searchable in S1_File. To address the reviewer’s comment, we have now added the following text on pg9 lines 258-264,:

Previous work demonstrates that serine-threonine phosphatases, including PP2A and Ste20 Kinases[(34,35)](https://www.zotero.org/google-docs/?eEkM7R), impact DLK signaling. We searched for differentially regulated serine-threonine kinases, but found few significant DLK-dependent changes after nocodazole treatment other than modest changes in the expression levels of the serine-threonine phosphatases Ppp4r4, Ppp1r15a and Ptprr (S1_File).

12. Reviewer 1(Minor Comment 3) suggests references are not consistently cited and hard to follow.

References have been formatted in Vancouver style consistent with PLOS One guidelines.

13. Reviewer 1(Minor Comment 4) notes that supplemental data is only cited in Methods.

Supplemental data contains information related to the quality of the RNAseq dataset, and so we felt that the methods were the appropriate spot.

14. Reviewer 1(Minor Comment 5) notes that in previous literature Rae1 does not lead to defects in axonal microtubule stability.

Previous literature demonstrates that loss of Rae1 impacts levels of the E3 ubiquitin-ligase, Highwire, as reflected on pg 7 lines (229-233). In other contexts, Rae1 has been shown to regulate microtubule stability. It is not known if Rae1 directly impacts axonal microtubule stability. Language in the introduction has now been changed to reflect this ambiguity in the role of Rae1 in the axon. Line 51 has been altered to reflect this uncertainty about Rae1. As the discussion reflects the 1) identification of Rae1 as both a highwire regulator and 2) microtubule interactor, lines 229-233 have not been altered.

We have added the following references to identify Rae1 as microtubule regulator:

[45.	Blower MD, Nachury M, Heald R, Weis K. A Rae1-containing ribonucleoprotein complex is required for mitotic spindle assembly. Cell. 2005 Apr 22;121(2):223–34.](https://www.zotero.org/google-docs/?EFTUZw)

[46. Wong RW, Blobel G, Coutavas E. Rae1 interaction with NuMA is required for bipolar spindle formation. Proc Natl Acad Sci U S A. 2006 Dec 26;103(52):19783–7.](https://www.zotero.org/google-docs/?EFTUZw)

15. Reviewer 2 (Comment 1) notes that peripheral neurons have been shown to return to a basal state after injury, but other neurons, including central neurons, have not been shown to return to a basal state after injury.

Thank you for the suggestion, pg. 2, line 63 has been changed to reflect that *in vitro* peripheral neurons return to a basal state. PMID: [25447942](https://pubmed.ncbi.nlm.nih.gov/25447942) uses data from adult-derived neurons, suggesting this phenomenon is not limited to immature neurons.

16. Reviewer 2 (Comment 3) notes that the fixation protocol for tubulin in Fig 1F may not be able to distinguish between microtubules and free tubulin.

Thank you for the suggestion. To address this concern, we have performed an additional immunohistochemistry optimized for microtubule preservation. To preserve the cytoskeleton, we used a cytoskeleton extraction buffer, followed 1% glutaraldehyde fixative and 0.1% sodium borohydride reduction. We subsequently stained for detyrosined microtubules, a marker of stable microtubule populations in axons. We did not observe the fragmentation phenotype seen in Tuj1 staining after PFA fixation. We have amended Fig 1 and our discussion of Figure 1 results to reflect these results, significantly toning down our conclusions. The text on pg8 lines 220-235 has been changed to:

Since nocodazole inhibits microtubule polymerization, we examined the impact on microtubule integrity. As predicted from prior studies which use low dose of nocodazole [(28)](https://www.zotero.org/google-docs/?P5RMYs), microtubule integrity was maintained in control and nocodazole treated neurons. To test for changes in microtubule distribution, we stained for the neuronal tubulin marker, Tubb3, Tuj1 (Fig 1F,G). Loss of Tubb3 has previously been shown to lead to microtubule regrowth defects in DRGs *in vivo* [(31, 32)](https://www.zotero.org/google-docs/?hz2Rt8). To determine the DLK dependence of Tubb3 distribution, we evaluated the effect of DLK inhibitor on neurons treated with DMSO (control) and nocodazole. We observed no defects in DLK inhibitor treated neurons. However, DLK inhibition in nocodazole-treated neurons causes defects in Tuj1 staining, including gapping within the microtubules leading to a bead-on-a-string appearance along the axon. Quantification of microtubule continuity demonstrates a significant defect upon DLK inhibition in nocodazole-treated neurons (Fig 1F,G). This suggests that DLK signaling is required to maintain Tubb3 across axons, potentially facilitating microtubule regrowth and repair. To complement these experiments, we asked if the distribution of stable microtubule populations changed upon DLK signaling perturbation using a microtubule preservation protocol [(33)](https://www.zotero.org/google-docs/?L8eQns) to stain for detyrosinated microtubule populations. We observed continuous detyrosinated tubulin staining across all conditions. DLK inhibition in nocodazole-treated neurons led to circular microtubule structures after treatment (Fig 1H), suggesting some abnormalities, but not the frank fragmentation suggested by the Tuj1 staining. We next investigated if transcriptional changes could contribute to these alterations in axon morphology.

[31.	Latremoliere A, Cheng L, DeLisle M, Wu C, Chew S, Hutchinson EB, et al. Neuronal-Specific TUBB3 Is Not Required for Normal Neuronal Function but Is Essential for Timely Axon Regeneration. Cell Rep. 2018 Aug 14;24(7):1865-1879.e9.](https://www.zotero.org/google-docs/?EFTUZw)

[32.	Radwitz J, Hausrat TJ, Heisler FF, Janiesch PC, Pechmann Y, Rübhausen M, et al. Tubb3 expression levels are sensitive to neuronal activity changes and determine microtubule growth and kinesin-mediated transport. Cell Mol Life Sci. 2022 Oct 29;79(11):575.](https://www.zotero.org/google-docs/?EFTUZw)

[33.	Zhang C, Kang JS, Asano SM, Gao R, Boyden ES. Expansion Microscopy for Beginners: Visualizing Microtubules in Expanded Cultured HeLa Cells. Curr Protoc Neurosci. 2020;92(1):e96.](https://www.zotero.org/google-docs/?EFTUZw)

To tone down our text, we have removed claims about microtubule fragmentation. This modified text includes p14 lines 409-411:

We demonstrate that DLK is required for alteration of the axonal actin and microtubule cytoskeleton after perturbation with nocodazole. While we did not prove that this effect is due to the expression of DLK-dependent genes, this finding is consistent with the model that a DLK-dependent mechanism alters the cytoskeleton.

Page 2, line 60 and page 13, line 377, which both refer the criteria for the homeostatic network:

(5) changes to the cytoskeleton.

Abstract:

Genetic and pharmacological perturbation of the cytoskeleton enhances the regenerative potential of neurons. This response requires Dual-leucine Zipper Kinase (DLK), a neuronal stress sensor that is a central regulator of axon regeneration and degeneration. The damage and repair aspects of this response are reminiscent of other cellular homeostatic systems, suggesting that a cytoskeletal homeostatic response exists. In this study, we propose a framework for understanding DLK mediated neuronal cytoskeletal homeostasis. We demonstrate that low dose nocodazole treatment activates DLK signaling Activation of DLK signaling results in a DLK-dependent transcriptional signature, which we identify through RNA-seq. This signature includes genes likely to attenuate DLK signaling while simultaneously inducing actin regulating genes. We identify alterations to the cytoskeleton including actin-based morphological changes to the axon. These results are consistent with the model that cytoskeletal disruption in the neuron induces a DLK-dependent homeostatic mechanism, which we term the Cytoskeletal Stress Response (CSR) pathway.

17. Reviewer 2 (Comment 4) notes that nocodazole stability may decline during the course of treatment and suggests this possibility should be addressed in the Discussion.

We thank the reviewers for this comment and agree that the concentration of nocodazole may decline over 16 hours. However, we note that changes in nocodazole concentration are internally controlled within our experiment (ie DMSO, Noc v DLKi, Noc). We have changed our initial description of the experiment on pg 6, line 173 to reflect this:

While nocodazole concentration may decline over the course of 16 hours, concentration will be consistent within experimental groups.

18. Reviewer 2 (Comment 5) notes that injury and trophic deprivation protocols both activate DLK signaling cascades, yet produce dramatically different outcomes (regeneration, cell death). These insults produce similar transcriptional signatures, including DUSP upregulation. This suggests that DUSP upregulation may not sufficiently explain why nocodazole treatment enables neurons to regenerate, while trophic deprivation facilitates cell death. Discussion of DUSP expression should be reworded in light of this.

This is an important comment and we’ve added the following to our initial description of the transcriptional comparison on pg 12, lines 340-343:

These results suggest that DLK activation, either through low dose nocodazole treatment or NGF deprivation, results in a shared transcriptional signature. Despite this shared transcriptional signature, treatment with low dose nocodazole or NGF deprivation results in different neuronal phenotypes, enhanced growth or cell death, suggesting that the impact of the transcriptional signature is context dependent.

PLOS ONE additionally notes the following changes should be made:

1. Manuscript should be formatted according to PLOS ONE style requirements
2. The following statement should be added "The funders had no role in study design, data collection and analysis, decision to publish, or preparation of the manuscript."
3. Funding statements are not consistent between the Funding section and Acknowledgement section. Currently, your Funding Statement reads as follows: "LD: F32NS117784 (national institute of neurological disorders and stroke) AD, JM: R01NS087632 (national institute of neurological disorders and stroke) AD: R37NS065053 (national institute of neurological disorders and stroke) MB: GM 141012 (national institute general medical sciences)"
4. The following statement should be added to the cover letter: "This does not alter our adherence to PLOS ONE policies on sharing data and materials.”
5. Please upload the minimal data set to reach the conclusions drawn in the manuscript.
6. PLOS ONE requires original and unadjusted images underlying all blot or gel results in the supporting information files.
7. Please include captions for Supporting information.

We have made all requested editorial changes.
